# GediNET for discovering gene associations across diseases using knowledge based machine learning approach

**DOI:** 10.1038/s41598-022-24421-0

**Published:** 2022-11-19

**Authors:** Emma Qumsiyeh, Louise Showe, Malik Yousef

**Affiliations:** 1 Information Technology Engineering, Al-Quds University, Abu Dis, Palestine; 2 The Wistar Institute, Philadelphia, PA 19104 USA; 3 Department of Information Systems, Zefat Academic College, 13206 Zefat, Israel; 4 Galilee Digital Health Research Center (GDH), Zefat Academic College, Zefat, Israel

**Keywords:** Cancer, Computational biology and bioinformatics, Genetics, Molecular biology, Biomarkers, Diseases

## Abstract

The most common approaches to discovering genes associated with specific diseases are based on machine learning and use a variety of feature selection techniques to identify significant genes that can serve as biomarkers for a given disease. More recently, the integration in this process of prior knowledge-based approaches has shown significant promise in the discovery of new biomarkers with potential translational applications. In this study, we developed a novel approach, GediNET, that integrates prior biological knowledge to gene Groups that are shown to be associated with a specific disease such as a cancer. The novelty of GediNET is that it then also allows the discovery of significant associations between that specific disease and other diseases. The initial step in this process involves the identification of gene Groups. The Groups are then subjected to a Scoring component to identify the top performing classification Groups. The top-ranked gene Groups are then used to train a Machine Learning Model. The process of Grouping, Scoring and Modelling (G-S-M) is used by GediNET to identify other diseases that are similarly associated with this signature. GediNET identifies these relationships through Disease–Disease Association (DDA) based machine learning. DDA explores novel associations between diseases and identifies relationships which could be used to further improve approaches to diagnosis, prognosis, and treatment. The GediNET KNIME workflow can be downloaded from: https://github.com/malikyousef/GediNET.git or https://kni.me/w/3kH1SQV_mMUsMTS.

## Introduction

Complex diseases like diabetes, Alzheimer’s, and cancer are influenced by genetics, lifestyle, and environmental factors and do not follow any clear inheritance patterns. Research targeting gene expression patterns seeks identify disease associated genes that can potentially be used to identify biomarker patterns associated with early diagnosis, prognosis, and development of an effective drug design^1^. Biomarker identification and sample classification, has become an attractive research area in the field of bioinformatics^2–5^.

Over the last decade, the availability of large datasets has contributed to forming rich data repositories such as miRTarBase^6^ for microRNA target genes, Gene Ontology (GO)^7^, Gene Expression Omnibus (GEO), which provides access to microarray measurements^8^, TCGA—a database for gene expression, RNA-seq^9^, and KEGG—a knowledge-base of pathways^10^. Another widely used biological resource is DisGeNET, a knowledge-based platform for gene-disease–variant associations^11^. Researchers can leverage these resources for in-silico validation and to train statistical machine learning models for classification and biomarker discovery.

Hallmarks of human diseases include the critical perturbation in gene(s)/protein(s) in critical molecular pathways that can produce divergent or lethal phenotypes. This “principle of guilt-by-association” suggests that associated genes can share functions through genetic or physical interactions^12^. In other words, genes responsible for similar diseases/phenotypes are likely to be similar. This finding has motivated a shift from the traditional pure data-oriented approaches to knowledge-based integrative approaches. Insights can be better attained when advanced tools exploit biological knowledge for deep analysis rather than just using the traditional clustering and machine learning approaches^13,14^.

Different studies identifying genes associated with human diseases have resulted in the development of tools for diagnosis and, in some cases, have led to the design of novel drugs. Many computational tools that differ in their approaches and use of resources have been described, including those that integrate various types of biological information into machine learning^15,16^. One integrative approach is to use the aggregation of multiple datasets to increase the statistical power to effectively identify a small subset of genes to predict disease types^17^. BioGraph, presented by Liekens et al.^18^ is a data-mining platform for disease gene prioritization and identification that integrates 21 curated biomedical databases in order to rank disease-gene relations and identify potential susceptibility genes. Other approaches, such as GeP-HMRF integrate Genome-wide association studies (GWAS), expression quantitative trait loci (eQTL), and protein–protein interaction (PPI) data^19^. GeP-HMRF is a unified statistical model to predict disease-related genes that is reported to outperform Sherlock^20^, COLOC^21^, and NetWAS^22^ tools. The work of Peng et al.^23^ proposes a new network-based disease gene prediction method called SLN-SRW (Simplified Laplacian Normalization-Supervised Random Walk) to generate edge weights of a new biomedical network by integrating heterogeneous sources of biomedical data.

The study by Asif et al.2018^16^ demonstrated that machine learning classifiers trained on functional gene similarities, using Gene Ontology (GO) to compute similarities between genes improves the identification of genes involved in complex diseases such as autism spectrum disorder (ASD). Luo et al.^24^ proposed EdgCSN, an ensemble learning algorithm that uses protein–protein interaction networks extracted from clinical sample-based networks, to predict disease-associated genes.

DisGeNET is a database^11^ that includes a variety of data for different diseases. Hamzeh and Rueda have proposed a new machine learning method incorporating the DisGeNET database to detect biomarkers in prostate cancer. A wrapper-based feature-selection approach was used to group genes-related diseases based on their classification accuracy. Results for each iteration were saved for further validation by researchers based on the best AUC or the highest number of detected genes in each group^11^.

Yousef et al. developed the Grouping-Scoring-Modeling (G-S-M) approach for integrating biological knowledge through different computational tools such as SVM-RCE-R^25,26^ maTE^27^, CogNet^28^, mirCorrnet^29^, miRModuleNet^30^, and PriPath^31^. Integrating biological knowledge with gene expression selection was reviewed in^38^ SVM-RCE-R^25,26^ tools were the first reports that considered groups of genes rather than individual genes, SVM-RCE (Support Vector Machines -Recursive Cluster Elimination), groups genes based on their gene expression values and scores each cluster of genes by a machine learning algorithm. In a recent study, Yousef et al.^32^, used the G-S-M model to integrate Gene Ontology data for grouping genes. In SVM-RNE (Recursive Network elimination)^33^ they detected gene networks that serve as gene groups for scoring and ranking by adopting the G-S-M model. Although different studies have used mRNA expression data and knowledge bases such as DisGeNet in their studies, our main objective using the G-S-M approach, has been to group genes to identify the best groups that were related to a specific disease. GediNET, our novel machine learning approach with two-class classification does not need other data annotations. With Monte Carlo cross-validation (MCCV), fractions of the samples are randomly selected as training dataset, and the rest is assigned for the testing dataset. The most accurate disease-gene groups are then identified in each training iteration, later accumulative top-ranked groups are combined to train the model. We also examined the results using similar approaches that follow the same merit, such as maTE^27^, CogNet^28^, mirCorrnet^29^, miRModuleNet^30^, and PriPath^31^.

However, the aim of the GediNET is not to compete with other tools that focus on single disease signatures but rather the aim is to discover novel gene groups with associations across a subset of disease based on machine learning.

## Materials and methods

All methods were performed in accordance with the relevant guidelines and regulations.

### Datasets

We downloaded 10 human gene expression datasets for different types of complex diseases from GEO database^8^. For each dataset, the name of the disease and the number of samples were defined. Moreover, positive and negative samples were available. Table 1 describes the 10 datasets in more detail.

**Table 1:** Description of the 10 datasets used in the study.

GEO accession	Title	Disease	#Samples	Classes
GDS1962	Glioma-derived stem cell factor effect on angiogenesis in the brain	Glioma	180	Negative = 23
				Positive = 157
GDS2545	Metastatic prostate cancer (HG-U95A)	Prostate cancer	171	Negative = 81
				Positive = 90
GDS2771	Large airway epithelial cells from cigarette smokers with suspect lung cancer	Lung cancer	192	Negative = 90
				Positive = 102
GDS3257	Cigarette smoking effect on lung adenocarcinoma	Lung adenocarcinoma	107	Negative = 49
				Positive = 58
GDS4206	Pediatric acute leukemia patients with early relapse: white blood cells	Leukemia	197	Negative = 157
				Positive = 40
GDS5499	Pulmonary hypertension: PBMCs	Pulmonary hypertension	140	Negative = 41
				Positive = 99
GDS3837	Non-small cell lung carcinoma in female nonsmokers	Lung cancer	120	Negative = 60
				Positive = 60
GDS4516_4718	Colorectal cancer: laser microdissected tumor tissues	Colorectal cancer	148	Negative = 44
				Positive = 104
GDS2547	Metastatic prostate cancer (HG-U95C)	Prostate cancer	164	Negative = 75
				Positive = 89
GDS3268	Colon epithelial biopsies of ulcerative colitis patients	Colitis	202	Negative = 73
				Positive = 129

Each entry has the GEO accession, the name of the disease, the number of samples and the data classes.

#### DisGeNET disease-gene association dataset

**Figure 1:**
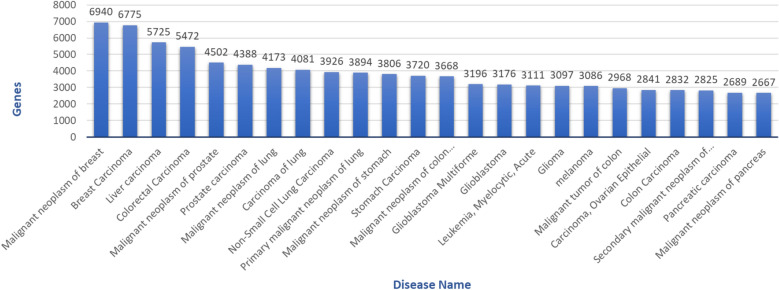
A part of the DisGeNET dataset histogram frequency plot. It shows the number of genes associated with each disease, where the X-axis is the disease name, and Y-axis is the number of genes.

The dataset containing genes and their associated diseases was downloaded from DisGeNET version 7.0^11^. The dataset contains 30,170 diseases and 21,666 genes that form 3,241,576 gene-disease connections. Given the massive dataset size, two filters were used to reduce the number of associations in terms of practicality and to reduce the computational complexity. The filters were set on the columns *diseaseType* and *diseaseSemanticType* in the DisGeNET dataset. The *diseaseType* column divided the data into three categories—disease, phenotype, and group—and we only chose disease as concerning for our study. On the column *diseaseSemanticType*, we only chose those rows categorized as *Neoplastic Process* and *Disease*. This was done to increase compatibility and to better understand the workflow results. After filtering, only 15,991 genes and 3929 diseases remained for further analysis, which accounted for 329,936 gene-disease associations. Figure 1 illustrates a part of the disease distribution over the number of genes for each disease.

### The merit of GediNET in the discovery of disease-disease associations

Let D be a two-class gene expression dataset designed to study a specific disease (for example, Lung Cancer or Breast cancer) in order to detect significant genes that will serve as a biomarker for distinguishing cancer vs non-cancer. The traditional approach of the classification model suggests a list of *k* genes that can serve as biomarkers for predicting those patients with the disease. In other words, identifying disease-gene associations. One possible solution could be a linear function F(X) that might be expressed as:

F(X) = *w*_*1*_*g*_*1*_ + *w*_*2*_*g*_*2*_ + *…* + *w*_*k*_*g*_*k*_, where *w*_*i*_ are the weights (scores) while the *g*_*i*_ are the gene expression values. The weights indicate the importance (significant) of each gene expression for the linear model. For instance, a value weight close to zero indicates that the associated genes contribute less to the equation model. In other words, F(X) describes the biological interaction between those *k* individual genes to form a biomarker signature.

GediNET differs from traditional approaches by considering groups of genes, rather than individual genes. A group is a disease name that represents pre-existing biological knowledge of the associations between sets of genes and the disease. GediNET scores those individual groups and their contribution to the classification task by applying the S component of GediNET (see section (The S component). The top j-scored genes groups will be used for training the final model of GediNET. In other words, the genes that appear on those j groups will be used to train the machine learning model. The S component relies on representing the gene groups as a sub-dataset of the original dataset D preserving the class labels, as described in detail in the two following sections (Grouping Genes based on Disease (The G component) and Creating a Sub-dataset).

**Figure 2:**
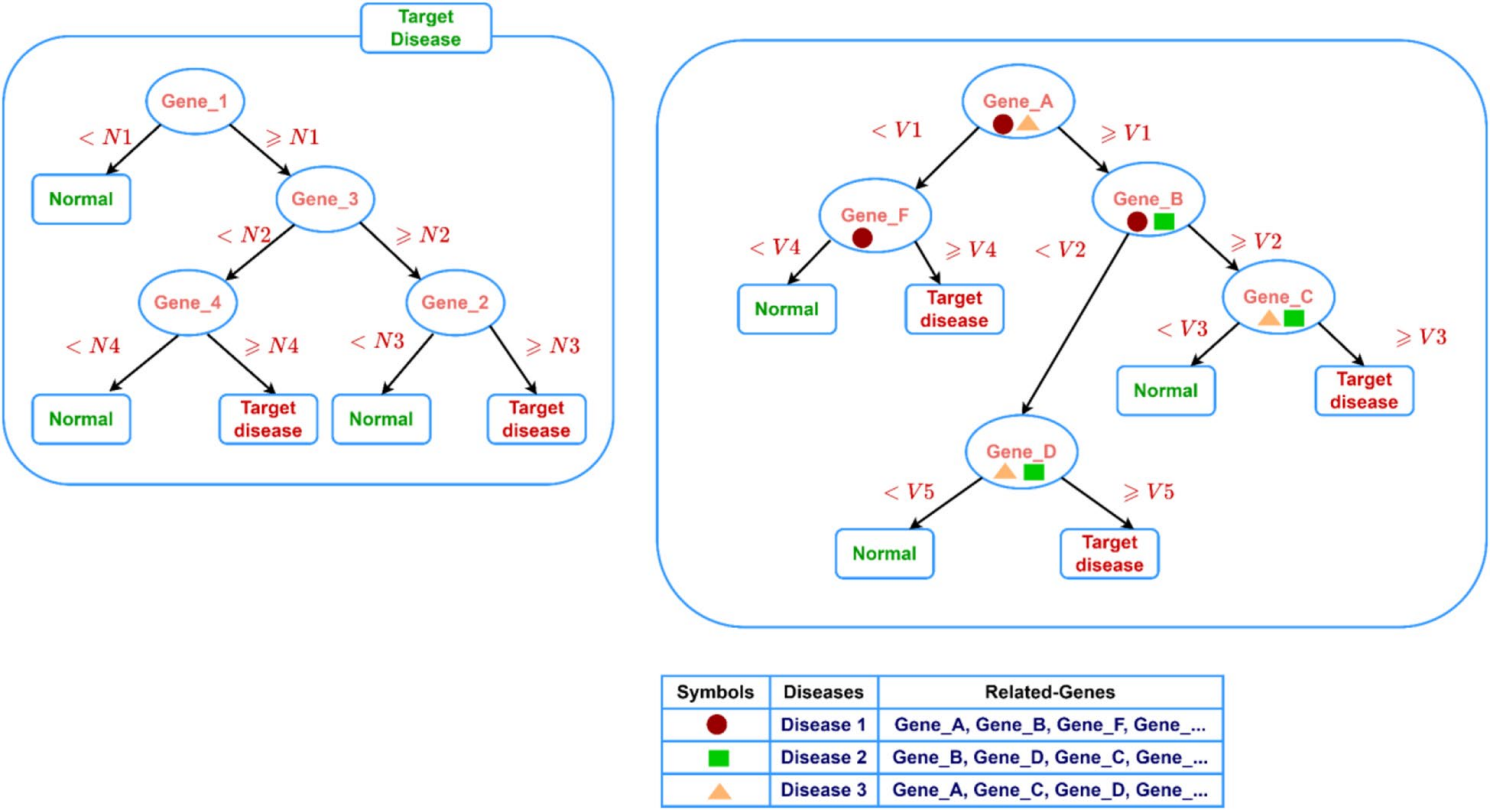
Decision Tree model. The left panel illustrates the traditional approach that detects gene-disease associations, while the right panel illustrates the disease-disease association as the output of GediNET.

For simplicity, the final model might be visualized as a decision tree, as illustrated in Fig. 2 (Right panel). The left panel of Fig. 2 illustrates the decision tree model of the significant genes selected by the traditional approach. The right panel of Fig. 2 shows that the decision tree model consists of genes associated with the top three GediNET ranked diseases (groups). This model contains information about biological knowledge of the diseases showing the disease-disease associations.

For example, considering the dataset GDS1962 that studies Glioma, GediNET suggests a model that is based on the top three significant groups/diseases, as follows:

$${\text{Grp1}}\_{\text{disease}} = \, \left\{ {\text{PAPILLARY RENAL CELL CARCINOMA}} \right\},{\text{ Grp2}}\_{\text{disease}} = \, \left\{ {\text{PLASMA CELL}} \right\},{\text{ and Grp3}}\_{\text{disease}} = \, \left\{ {\text{NEOPLASM and ADULT GLIOBLASTOMA}} \right\}.$$

The following are the sets of genes associated with each disease:

$${\text{Grp1}}\_{\text{genes}} = \, \left\{ {{\text{SLC16A1}},{\text{ TAGLN2}},{\text{ TIMP3}},{\text{ IGFBP7}},{\text{ TOP2A}},{\text{ TP53}},{\text{ RRM2}} \ldots } \right\},{\text{ Grp2}}\_{\text{ genes }} = \, \left\{ {{\text{CD99}},{\text{ TP53}},{\text{ LPL}},{\text{ CD4}}0,{\text{ CD38}},{\text{ NCAM1}},{\text{ MYC}},{\text{ CSF3}},{\text{ CDKN2A}},{\text{ FGFR3}},{\text{ CCND1}}} \right\},{\text{ and Grp3}}\_{\text{genes}} = \, \left\{ {{\text{EDNRA}},{\text{ CSPG4}},{\text{ MELK}},{\text{ ENPEP}}, \, \ldots } \right\}.$$

Applying GediNET will compute F*(x) that describes the association between the Grp 1, 2 and 3_diseases with the disease under study (in this case Glioma disease). This might lead to new discoveries that have not been observed before by traditional approaches.

### The G-S-M components of GediNET

**Figure 3:**
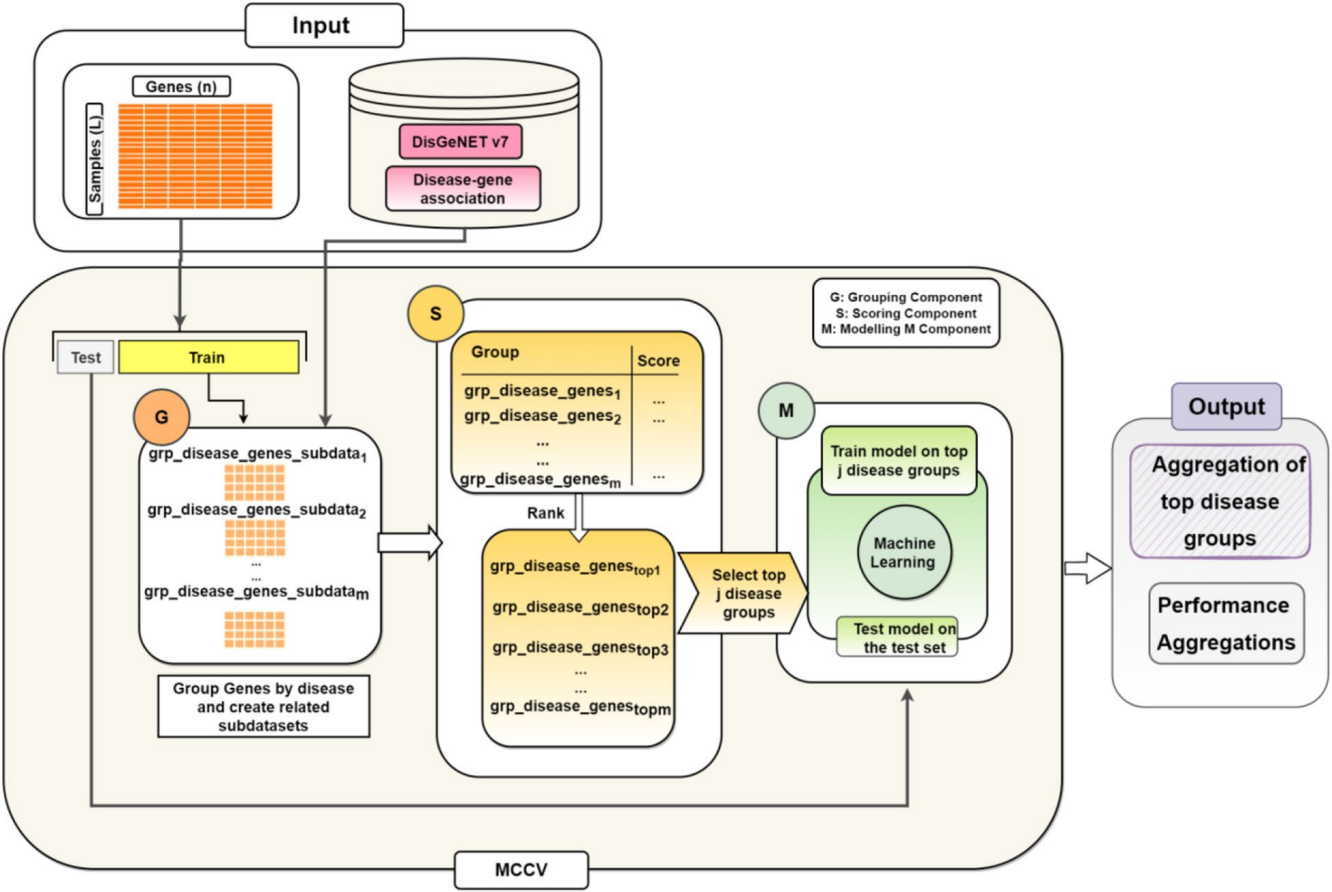
GediNET workflow. The main workflow of G-S-M that integrates pre-existing biological knowledge for grouping genes based on disease-gene association, which is derived from the DisGeNET v7 database.

GediNET is based on the generic approach named G-S-M, which has been adopted by different tools such as SVM-RCE ^34^, SVM-RCE-R^25^, SVM-RCE-R-OPT^26^, SVM-RNE^33^, maTE^27^, CogNet^28^ , miRcorrNet^29^, Integrating Gene Ontology-Based Grouping and Ranking^32^, miRModuleNet^30^, PriPath^31^ and recently reviewed in Yousef et al.^35^. The main workflow of GediNET is illustrated in Fig. 3, where the G-S-M approach is presented in the three main sections labeled with the orange section (G), the yellow section (S), and the green section (M), which represent:

- 1. The G Component (Grouping): where the genes are grouped according to the biological pre-existing knowledge of disease. Each group is represented by an extracted two-class subdataset from the main given dataset.
- 2. The S Component (Scoring): where the groups are scored and ranked by considering the related two-class subdatasets.
- 3. The M Component (Machine Learning model): where the model is created by training a classifier (Random Forest) on the top ranked groups’ genes.

The inputs for GediNET are a two-class gene expression dataset and a table that represents the biological pre-existing knowledge of the diseases. The dataset consists of two classes of samples: control (negative) and disease (positive). The dataset is split into training and testing. The training dataset is used for the G-S-M components, while the testing dataset is used to evaluate the model’s performance. The whole workflow is repeated 100 iterations using the cross-validation loop, where the input is randomly split into 90% training and 10% testing in each iteration. A Statistical *t* test (testing of equality of variances, Levene’s test)^36^ is performed on the training dataset to detect the top differentially expressed genes. The top 2000 differentially expressed genes with a P-value less than 0.05 are selected. The main contribution of the generic approach and the description of each component’s functions are explained in detail in the following sections.

### G component: grouping genes based on disease

The first component GediNET is the grouping component G (the orange section in Fig. 3), which separates genes into groups. The G component might be based on any pre-existing biological knowledge, such as miRTarBase, KEGG pathway, etc., for creating groups of genes. In this tool, the G component group genes based on the DisGeNET v7 database^11^, which are gene-disease associations. Table 2 is an example of such groups that includes the disease name (group name), the set of genes associated with this disease, and the last column is the number of genes in the associated group.

**Table 2:** An example of groups of diseases with their associated genes.

Group name	Genes	#Genes
Small cell carcinoma of lung	VPS13B, SLC16A1, ANXA1, CD99, SMARCC1, PCNA…	41
Leukemia, B-cell	TP53, LAMA4, STK11, CSPG4, CD40, TNFRSF1A…	43
Stage III breast cancer Ajcc V6	TP53, BRCA2	2
Head and neck carcinoma	PRMT5, ANXA1, LGALS1, TIMP3, IGFBP7, PCNA, TNC, TP53…	149
Secondary malignant neoplasm of bone	ADAM9, SLC16A1, CD99, NME1-NME2, DPYSL3, TNC, TP53, NRAS…	145
Malignant glioma	TK1, NPAS3, CD63, HMGB1, TAGLN2, TXNIP…	162
Adenocarcinoma, tubular	PCNA, TP53, EFEMP1, APOE, STK11, PRKD1…	31
Childhood brain neoplasm	TP53, NRAS, SOX9, MYC, TNFRSF11B	5
Adult myelodysplastic syndrome	CSNK1A1, CTNNA1, HMGB1, PCNA, TOP2A, TP53…	58
Non-small cell lung cancer stage I	TP53, PRRX1, IGFBP3, VEGFA, S100A6, GSTK1…	22

The last column represents the number of genes in each group (group size).

### G component: creating two-class subdataset

**Figure 4:**
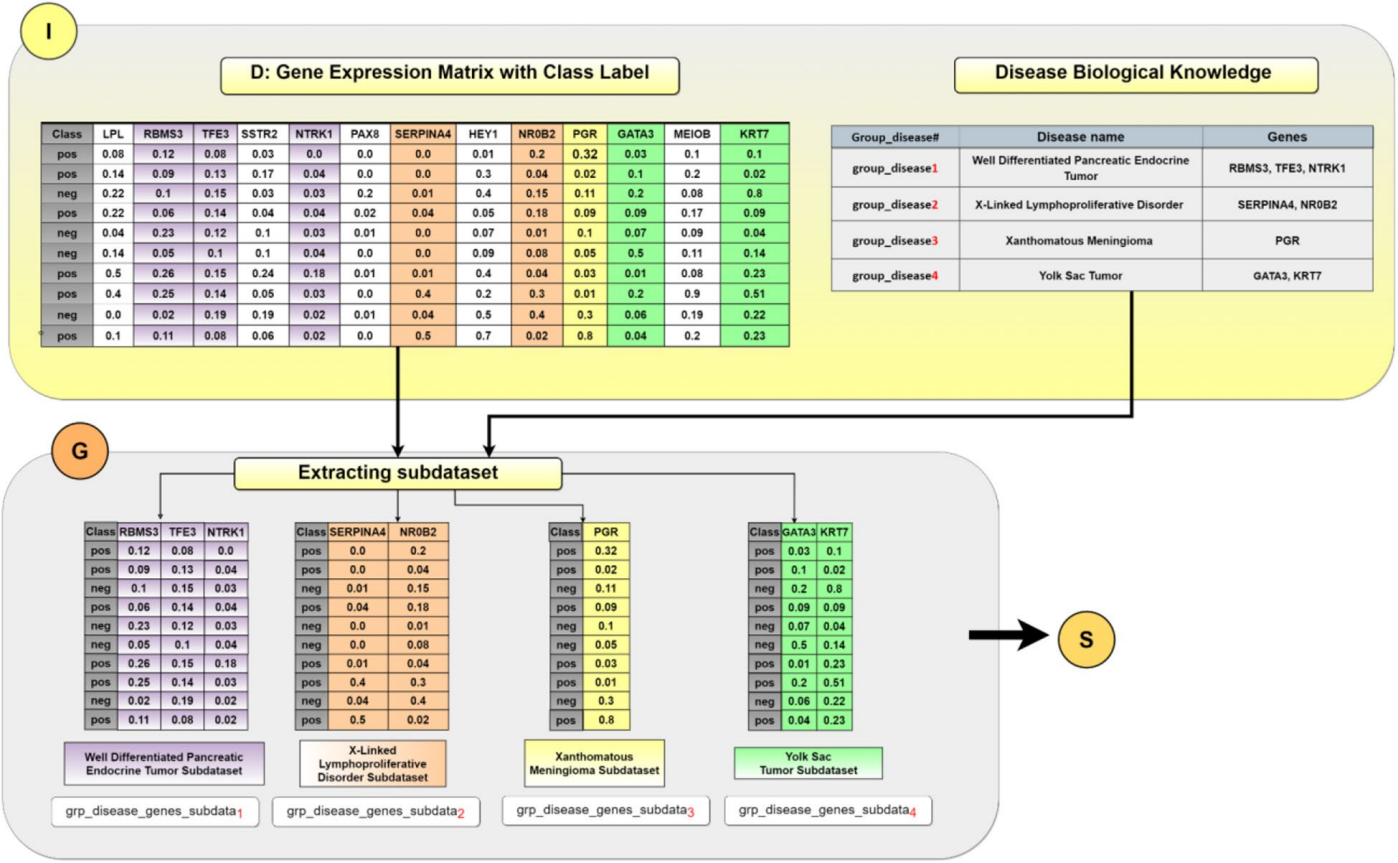
An example of creating two-class subdatasets extracted according to disease-group names. These subdatasets will be subject to the S component for scoring.

We assume that D consists of columns that represent the genes expressions while the rows represent the samples. D also has a class label column with information about each sample, as illustrated in Fig. 4 at the Input panel (labeled by I).

To score each group, we have created a two-class subdataset related to each group/disease. Each subdataset is specific for one group/disease that contains the genes belonging to that group/disease. This is achieved by extracting the genes columns belonging to the specific group and their original class label from the original dataset D. Let *m* be the number of groups. In this stage, we will extract or create *m* two-class subdatasets that will be input to the S (Scoring) component. In Fig. 4, the I panel (input panel) contains two matrices. The left one is an example of the gene expression matrix D with the class label for each sample appearing in column “Class”. The right one is the pre-existing biological knowledge containing the disease name (group name) with its set of genes. In our example, the right matrix contains four group diseases labeled with group_disease_i_, i = 1,…,4. For example, group_disease_1_ represents the disease named “*Well Differentiated Pancreatic Endocrine Tumor*,*”* along with three genes associated with this specific disease. The genes are RBMS3, TFE3, and NTRK1.

Within the G component, the extraction of two-class subdatasets is performed. As evident in Fig. 4, four subdatasets are created. For each subdataset, the gene columns belonging to each disease group are extracted from the D dataset with the original class label, where *pos* is for the positive class and *neg* for the negative class. The four subdatasets serve as input to the following component, S, to be scored and ranked.

### S component: scoring the groups

As a result of the G component, *m*, two-classes subdatasets are created, each representing one group. The task of the S component is to compute a score that measures to what extent it is differentially expressed considering the given two classes. The group is a set of genes; one way of computing a group-score is by computing each individual genes t statistics and then averaging those scores to be the final score of the group, as suggested in^37^. The following equations might be used to compute this score for given gene *i*:

1 $${T}_{i }= ({\mu }_{i\_pos}-{\mu }_{i\_neg})/\sqrt{\frac{{\sigma }_{{i}_{pos}}^{2}}{{n}_{1}}+\frac{{\sigma }_{{i}_{neg}}^{2}}{{n}_{0}},}$$

where ${\mu }_{i\_pos}$ and ${\mu }_{i\_neg}$ are the average expressions over the positive and negative class respectively. $\sigma_{ipos}$ and ${\sigma }_{ineg}$ are the standard deviations over the positive and negative class, while, ${n}_{1}$ is the number of positive class samples, and ${n}_{0}$ is the negative class samples.

Based on equation number 1, one might compute a score for a given group that consists of *k* genes as the following:

2 $$S(group) =\frac{1}{k}{\sum }_{i=1}^{k}{{T}_{i},}$$

**Figure 5:**
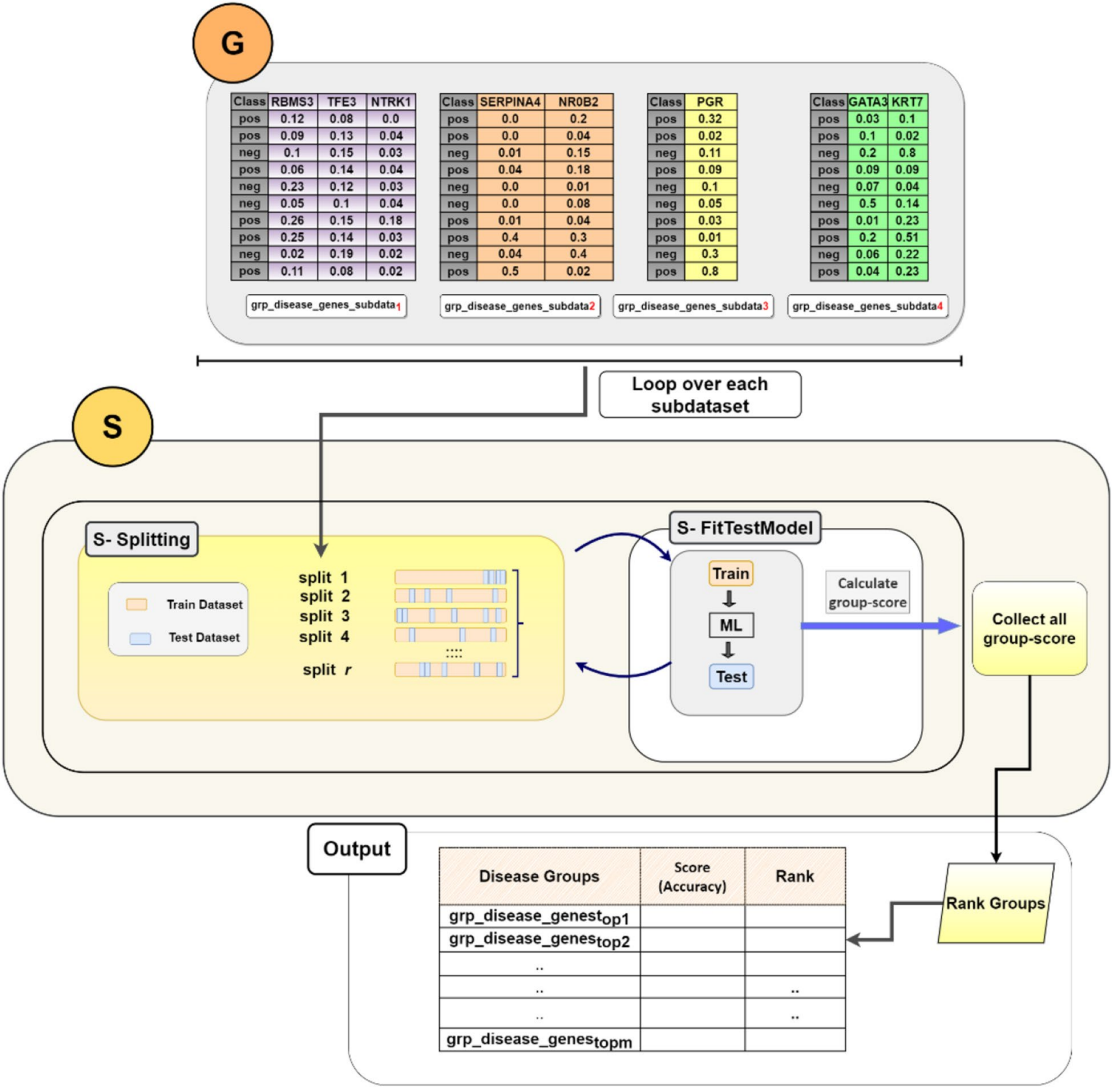
The details of the S component. The G panel contains all the two-class sub-datasets that each one is subject to the S component.

However, GediNET uses a more progressive approach based on machine learning to compute such scores. Figure 5 illustrates the steps of the S component that ends by assigning the performance measurement as the group score. In our case, we consider the accuracy. Each two-class subdataset is randomly split into training and testing (90% training and 10% testing) as shown in Fig. 5, Panel S-Splitting, where this procedure is repeated *r* times. The training is used to train the machine learning algorithm (we have used Random Forest), and the model’s performance is evaluated on the test split as seen in the Panel, S-FitTestModel. The accuracy average of the *r* splits is computed to form the group score. All of the group scores are collected to form a table of m scores. For the M component, we perform a ranking step by ordering the table in descending order. An example of such an output of the Scoring component applied to the GDS2545 dataset is presented in Table 3.

**Table 3:** An example of the output of the scoring S component. The first column is the name of the group disease, the Gene Set is the genes associated with the disease, the Score column is the computed score computed by the S component, and the Rank is the rank of the group based on the value of the score.

Disease	Genes set	Score	Rank
Papillary renal cell carcinoma	TP53, VEGFA, SNORD35B, …	0.98	1
Plasma cell neoplasm	LYN, IGF1, NME1, …	0.96	2
Adult glioblastoma	BRD2, DNMT1, MAOB, …	0.94	3
Intestinal cancer	CDKN2A, TP53, RPL24, …	0.91	4
Malignant neoplasm of colon stage IV	LARP1, PES1, IFI27, MEN1, …	0.89	5
Dermatofibrosarcoma	POSTN, AR, CDKN2A, TP53, …	0.87	6

GediNET uses the accuracy measurement to assign a score; one might use a different measurement or a combination of measurements (such as sensitivity, specificity, the Area under the curve, etc.). For more information on such an option, we refer to^26^.

### M component: fitting the model

The M component considers the top-ranked *j* groups of disease, and their genes are merged to form the top-ranked associated genes (as seen in Fig. 5, the output panel). A subdataset is extracted considering the top-ranked associated genes from the training part of the dataset (90% training, 10% testing, as mentioned before). An RF model is trained on the extracted subdataset. Finally, the model is evaluated on the testing dataset represented by those genes, and the performance statistics are recorded. We have reported the performance of *j* = 1,…,10.

In our implementation, many RF classifiers are trained on randomly selected data using 90% data for training and 10% for testing the classifier. However, such settings can be adjusted in our KNIME implementation of GediNET.

### Implementation of GediNET

We have implemented the GediNET tool using the free and open-source platform KNIME^38^ due to its simple and intuitive graphical user interface. KNIME is a highly integrative platform that has enabled the scope to utilize scripts in both python and R in tandem to implement our tool as a KNIME workflow.

The workflow created on KNIME comprises several nodes with their separate functions. Meta-nodes are created as a collection of nodes that perform specific tasks.

**Figure 6:**
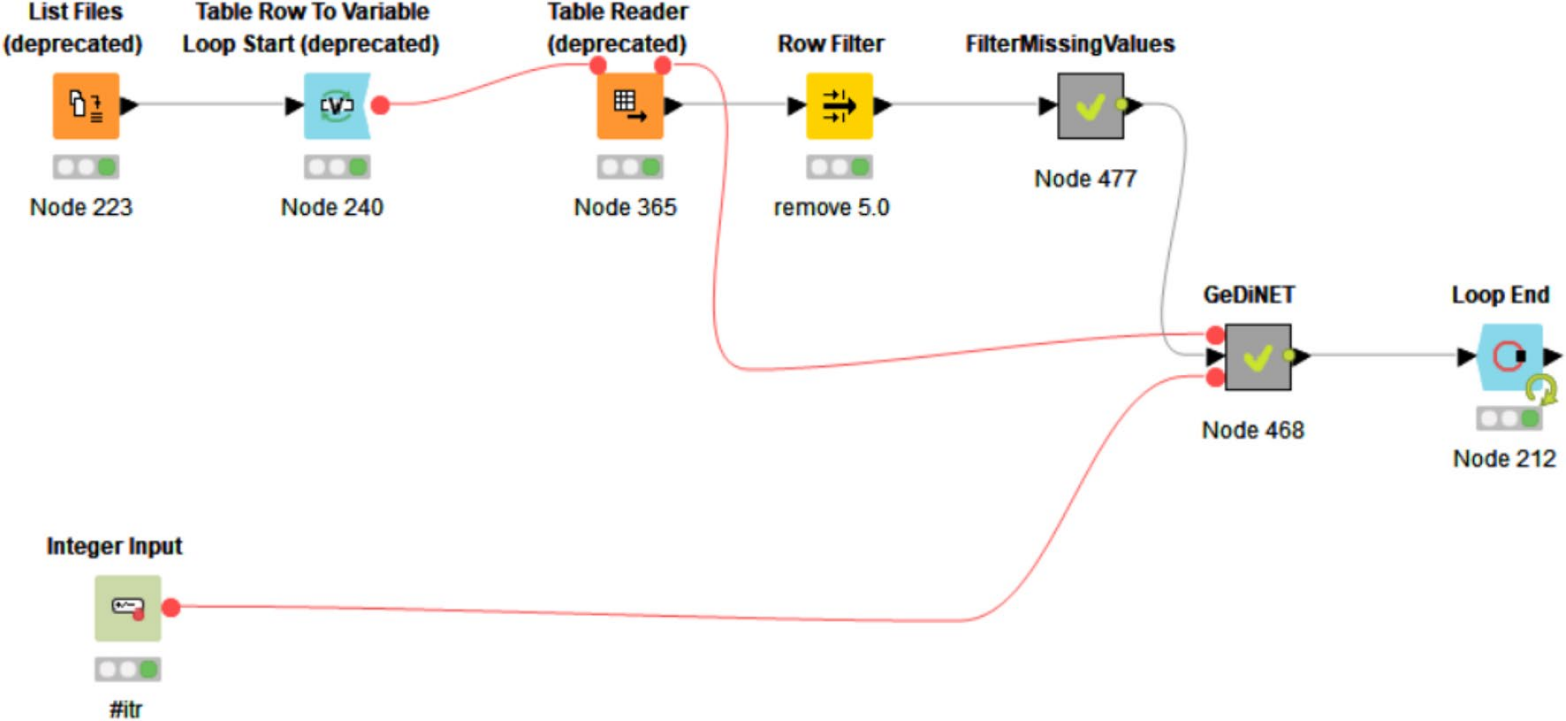
GediNET workflow in KNIME.

The KNIME workflow for GediNET is presented in Fig. 6. It starts by uploading a list of the names of the dataset via the “List Files/Folders” node. Then a loop over those datasets is run to read each dataset by the node “Table Reader”, which is then processed by the meta-node “FilterMissingValues” to remove and or filter out rows with missing values. It then sends the filtered data as input to the GediNET meta-node. While the “Integer Input” node allows modifying the number of iterations, the tool should be used while training the model.

The GediNET KNIME workflow could be downloaded from: https://github.com/malikyousef/GediNET or https://kni.me/w/3kH1SQV_mMUsMTS.

### Model performance evaluation

We used the Random Forest Classifier while splitting the data into 90% training and 10% testing. Since the datasets are imbalanced, meaning the dataset’s class label has an uneven distribution of observations, we employed the under-sampling method. Such a method deals with imbalanced datasets by maintaining all of the samples in the minority class while decreasing the size of the majority class. For model training, we applied tenfold Monte Carlo cross-validation (MCCV)^39^. With Monte Carlo cross-validation (MCCV), fractions of the samples are randomly selected as training data, and the rest is assigned for the test data. The performance measures are computed as the average of 100-fold MCCV. We use MCCV rather than traditional CV because the MCCV method is more repeatable since the variance is low.

To evaluate the performance of the RF model, several quantitative metrics were calculated, such as Accuracy, Sensitivity and Specificity^40^, using the following formulations:

3 $${\text{Sensitivity }}\left( {{\text{SEN}}} \right) \, = {\text{ TP}}/ \, \left( {{\text{TP }} + {\text{ FN}}} \right),$$

4 $${\text{Specificity }}\left( {{\text{SPE}}} \right) \, = {\text{ TN}}/ \, \left( {{\text{TN }} + {\text{ FP}}} \right),$$

5 $${\text{Accuracy }}\left( {{\text{ACC}}} \right) \, = \, \left( {{\text{TP }} + {\text{ TN}}} \right)/ \, \left( {{\text{TP }} + {\text{ TN }} + {\text{ FP }} + {\text{ FN}}} \right),$$

where TP = true positive; FP = false positive, TN = true negative; and FN = false negative. Moreover, the Area Under the Curve (AUC) measures the ability of a classifier to distinguish between classes and is used as a summary of the ROC curve^41^. We used the AUC to evaluate the performance results.

In each iteration, our approach generates lists of disease groups and their associated genes that are slightly different. Hence, there is a need to apply a prioritization approach on those lists. As utilized in miRcorrNet, we have used rank aggregation methods. In this respect, we have embedded the RobustRankAggreg R package^42^, developed by (Kolde et al.^42^), into the GediNET workflow. The RobustRankAggreg assigns a P-Value to each element in the aggregated list, which describes how well each element/entity was ranked compared to the expected value.

## Results

### Performance evaluation of GediNET

Table 4 presents an example of the average 100-fold MCCV performance table of GediNET for aggregated top-ranked 10 groups for the GDS1962 dataset. The last row presents the performance of the top-ranked group (#Groups = 1). The AUC obtained is 97% using 21.61 genes on average. The row of #Groups = 2 presents the performance metrics obtained for the top 2 groups, where the genes of the first top-ranked group and the second-highest scoring group are aggregated together. That is to say that GediNET reports the performance results for the top 10 groups cumulatively.

**Table 4:** An example averages of 100 MCCV performance table of GediNET for top-ranked 10 groups for GDS1962 dataset cumulatively.

#Groups	#Genes	Accuracy	Sensitivity	Specificity	AUC
10	136.74	0.928	0.93	0.92	0.98
9	127.68	0.93	0.93	0.92	0.98
8	116.02	0.93	0.94	0.92	0.98
7	111.16	0.93	0.93	0.91	0.98
6	102.02	0.93	0.9	0.92	0.98
5	92.88	0.93	0.93	0.93	0.98
4	78.37	0.93	0.93	0.92	0.98
3	62.47	0.93	0.94	0.92	0.98
2	45.57	0.93	0.93	0.93	0.97
1	21.61	0.92	0.93	0.92	0.97

**Figure 7:**
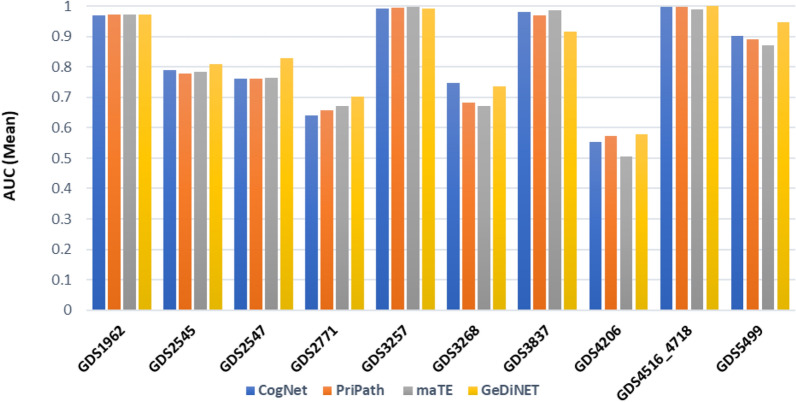
The mean AUC values of GediNET, CogNet, maTE and PriPath for ten different datasets for the top two groups.

Table 5 shows the GediNET performance over 10 datasets for the top 2 gene groups. All values are the results of an average of 100-MCCV iterations while considering the AUC for presenting the performance. The complete performance results are attached in the supplementary data. The table shows the GEO accession in the first column, the number of genes in column #Genes while ACC is the accuracy, SEN is the sensitivity, SPE is the specificity, and the AUC is the area under the curve. We see only one unsuccessful result for the dataset GDS4206. However, a similar observation was made when applying other tools to this specific dataset, as illustrated in Fig. 7.

**Table 5:** Performance results of GediNET over the top-ranked group.

GEO Accession	#Genes	ACC	SEN	SPE	AUC
GDS1962	45.57	0.93	0.93	0.93	0.97
GDS2545	113.76	0.73	0.72	0.74	0.81
GDS2771	97.83	0.64	0.69	0.59	0.70
GDS3257	74.81	0.97	0.99	0.94	0.99
GDS3837	21	0.92	0.83	1	0.92
GDS4206	83	0.66	0.3	0.82	0.58
GDS4516_4718	40.72	0.99	0.99	0.99	1
GDS2574	102.49	0.76	0.77	0.76	0.83
GDS3268	115.7	0.67	0.7	0.63	0.73
GDS5499	80.23	0.9	0.96	0.77	0.95

*ACC* accuracy, *SEN* sensitivity, *SPE* specificity, *FM* F-measure, *AUC* area under the ROC curve.

The average number of genes associated with the top 2 groups is slightly high because the distribution of genes over the disease is slightly high compared, for example, to other biological knowledge such as microRNA target or KEGG pathways. Moreover, this number of genes could be reduced by removing the least contributed genes when processing each group. This step will be considered in the future version of the algorithm. Also, one can use additional biological knowledge to filter out more genes from the group by, for example, leaving the most associated genes with the disease. The last suggestion requires other biological resources to be embedded into the GediNET.

### Comparative evaluation with other biological G-S-M

For comparison, we have considered similar tools that apply the G-S-M approach by integrating biological knowledge for grouping the genes and performing the scoring on the group, such as CogNet^30^, maTE^29^, and PriPath^33^ use RF with the same default parameters (Split criteria: Information Gain Ratio and number of models 100). Moreover, a similar approach was applied in the text mining domain where a TextNetTopics tool was developed^43^. Within the TextNetTopics, a performance comparison was performed with three different feature selection methods namely Extreme Gradient Boosting (XGBoost), Fast Correlation Based Filter (FCBF), and selectKBest (SKB), through four classifiers. These classifiers are Adaboost, DT, RF, and LogitBoost. The results showed that RF with SKB feature selection provided the highest performance.

We have recorded the AUC values for the top 1–10 groups ranked by the scoring component for each tool by applying 100-MCCV. More specifically, we considered the top two groups for comparison purposes.

**Figure 8:**
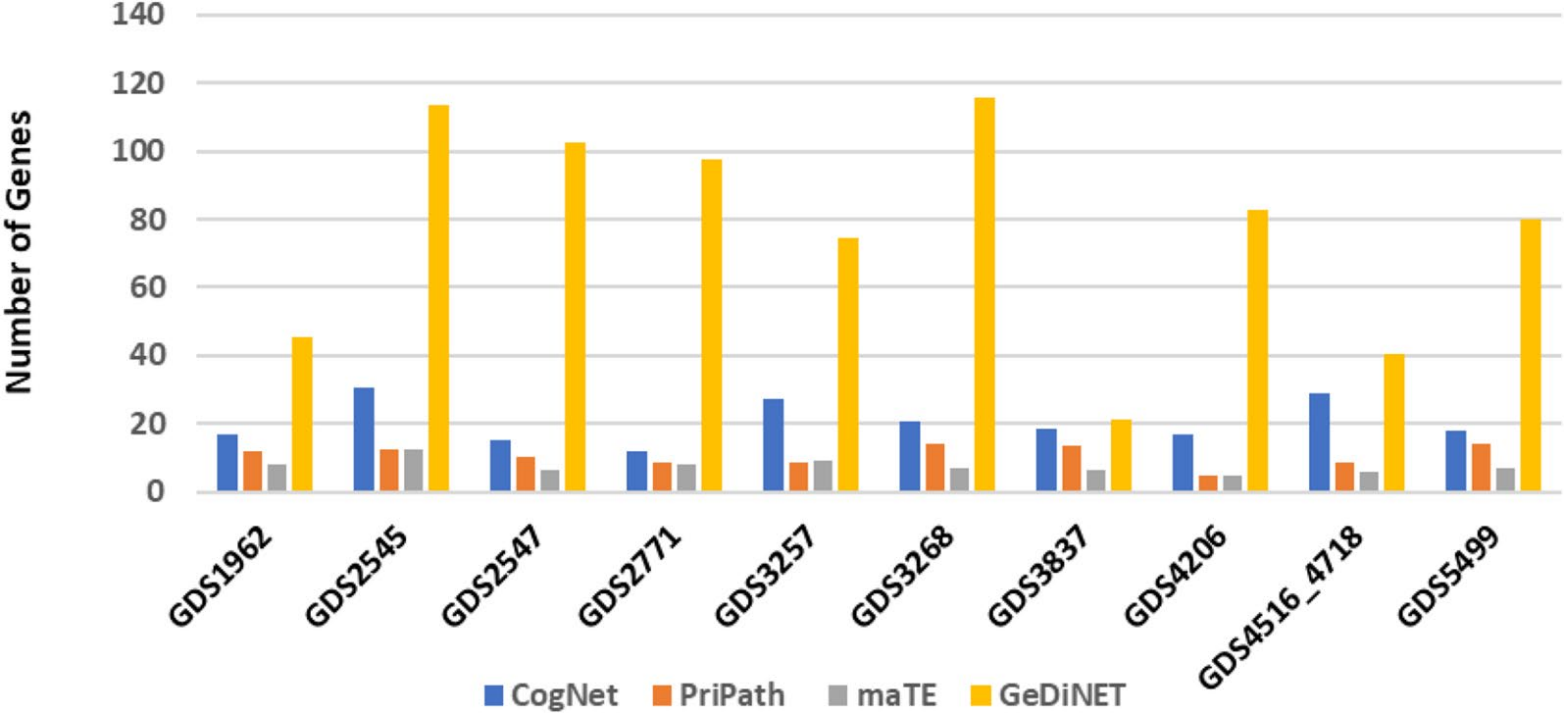
The mean number of genes of GediNET, CogNet, maTE and PriPath tools for ten different datasets for the top two groups.

Figure 7 illustrates the mean AUC values of the four tools for the 10 datasets. Meanwhile, Fig. 8 plots the mean number of genes for the four tools. As apparent in Fig. 7, the AUC values of GediNET, CogNet, maTE, and PriPath for 10 different datasets for the top two clusters are nearly similar. Thus, the performance of those tools is comparable. This close performance indicates that the developed tool GediNET is consistent and robust. However, the outcome of each tool is different as each one of those tools has its merit and its aim of detecting significant groups related to specific pre-biological knowledge.

Figure 8 implies that, on average, GediNET uses a tenfold higher number of genes than other tools. This is due to the fact that the groups of genes associated with the diseases are much higher than others.

One of the tool’s outputs is a list of ranked disease groups that were assigned a P-value by the robust rank aggregation package^42^. Table 6 is an example of this tool for the GDS1962 dataset.

**Table 6:** An output of the RobustRankAggreg tool for the GDS1962.

GDS1962			
Disease name	P-value	#Genes	List of genes
Papillary renal cell carcinoma	0.00052	22	SLC16A1, TAGLN2, TIMP3, IGFBP7…
Plasma cell neoplasm	0.0010	11	CD99, TP53, LPL, CD40…
Common acute lymphoblastic leukemia	0.001772	3	KNG1, MME, BCL2
Ductal breast carcinoma	0.002363	13	TCF21, AFAP1L2, PLG…
Gastric mucosa-associated lymphoid tissue lymphoma	0.002953	2	BCL2, EPCAM
Intrahepatic cholangiocarcinoma	0.003544	27	SHBG, BAX, TYMS, GPC3…
Lymphoma, non-hodgkin	0.004135	44	BAX, SLC23A1, MME, TYMS, …
Malignant neoplasm of colon stage iv	0.004725	7	TYMS, MYCN, KLK6, NDRG1, …
Neuroectodermal tumor, primitive	0.005316	14	SFRP1, PCSK2, MYCN, CAPS…
Papillary thyroid carcinoma	0.005907	75	BAX, PKHD1L1, MME, GPC3…

This is a novel output of the feature selection techniques that GediNET is providing. This table will be used to analyze the relationship between the diseases further. For example, Table 6 raises a biological question about the association between the top-ranked diseases (PAPILLARY RENAL CELL CARCINOMA, PLASMA CELL NEOPLASM,…) and the target disease of the study (dataset GDS1962 with target disease Glioma). Additionally, GediNET provides a list of significant genes that were also aggregated by the Robust Rank Aggregation tool. While scoring each group, the genes associated with the group is scored with the same score as the group. This list with its scores is aggregated at the end to compile and report a list of significant genes. Table 7 provides an example of such a list.

**Table 7:** Top 10 significant genes that were aggregated by the RobustRankAggreg tool for the GDS2545 dataset.

Genes	P-value
MYL1	0.003
RNF44	0.016
UBN1	0.051
N4BP2L1	0.060
GDI1	0.066
ARL17B	0.093
MYLPF	0.133

The user can consider the list of significant genes for functional and enrichment analysis as was done in similar studies such as PriPath and miRmodulnet using different tools such as David^44^, EnrichR^45^, and GeneMANIA^46^.

### Biological interpretations

One of the outputs of GediNET is a list of significant diseases which had been scored by the S component, as illustrated in Table 6. This list is ranked by P-value (ranked by RobustRankAggreg).

For all the 10 GEO datasets, the top 2 diseases and their set of genes were considered to perform pathway enrichment analysis. Their total number of distinct genes is 1184.

The web tool, EnrichR^45^ was used to perform the pathway enrichment analysis. The tool was run to collect the top enriched pathways for each disease-gene group per dataset, and the top pathways (with the least P-values) were selected. WikiPathway database^47^ version 2021 for human genes was used to select our results. The top cell signaling pathways’ names for the 10 GEO datasets, P-values, adjusted P-value, and associated genes are illustrated in Table 8. Evidence from literature was then gathered for the dataset cancer and the top-performing disease, along with the enriched genes and pathways found from the enrichment analysis.

**Table 8:** The top cell signaling pathways’ names for the 10 GEO datasets.

Cell signaling pathways term	P-value	Adjusted P-value	List of genes	#Genes
Head and neck squamous cell carcinoma WP4674	2.24E-13	6.31E-11	CCND1; CDKN2A; AKT1…	9
DNA damage response (only ATM dependent) WP710	2.95E-16	1.08E-13	GSK3B; SMAD4; CDKN1A,…	14
VEGFA-VEGFR2 signaling pathway WP3888	1.66E-10	6.37E-08	LRRC59; NRP2; PRKAA2;…	27
VEGFA-VEGFR2 signaling pathway WP3888	1.05E-11	2.59E-09	HSP90AA1; ANXA1;…	18
Lung fibrosis WP3624	6.32E-09	1.73E-06	GREM1; CSF3;IL6; PLAU; EGF; MUC5B; MMP9	7
IL-18 signaling pathway WP4754	2.33E-17	1.05E-14	GSK3B; CEBPB; CXCL8;…	29
Effects of nitric oxide WP1995	2.93E-05	0.00310457	NOS1; XDH	2
TP53 network WP1742	2.14E-13	9.13E-11	CDKN1A; CDKN2A; MYC;…	9
Apoptosis WP254	1.88E-06	4.25E-04	CASP10; MYC; PMAIP1;…	6
Hepatitis C and hepatocellular carcinoma WP3646	5.41E-12	2.07E-09	CDKN1A; IL6; CXCL8;…	10

The first column is the name of the cell signaling pathway, the second column is the P-values, the third column is the adjusted P-value, the Genes column represents an example of the associated genes, and finally, the last column is the total number of associated genes.

**Figure 9:**
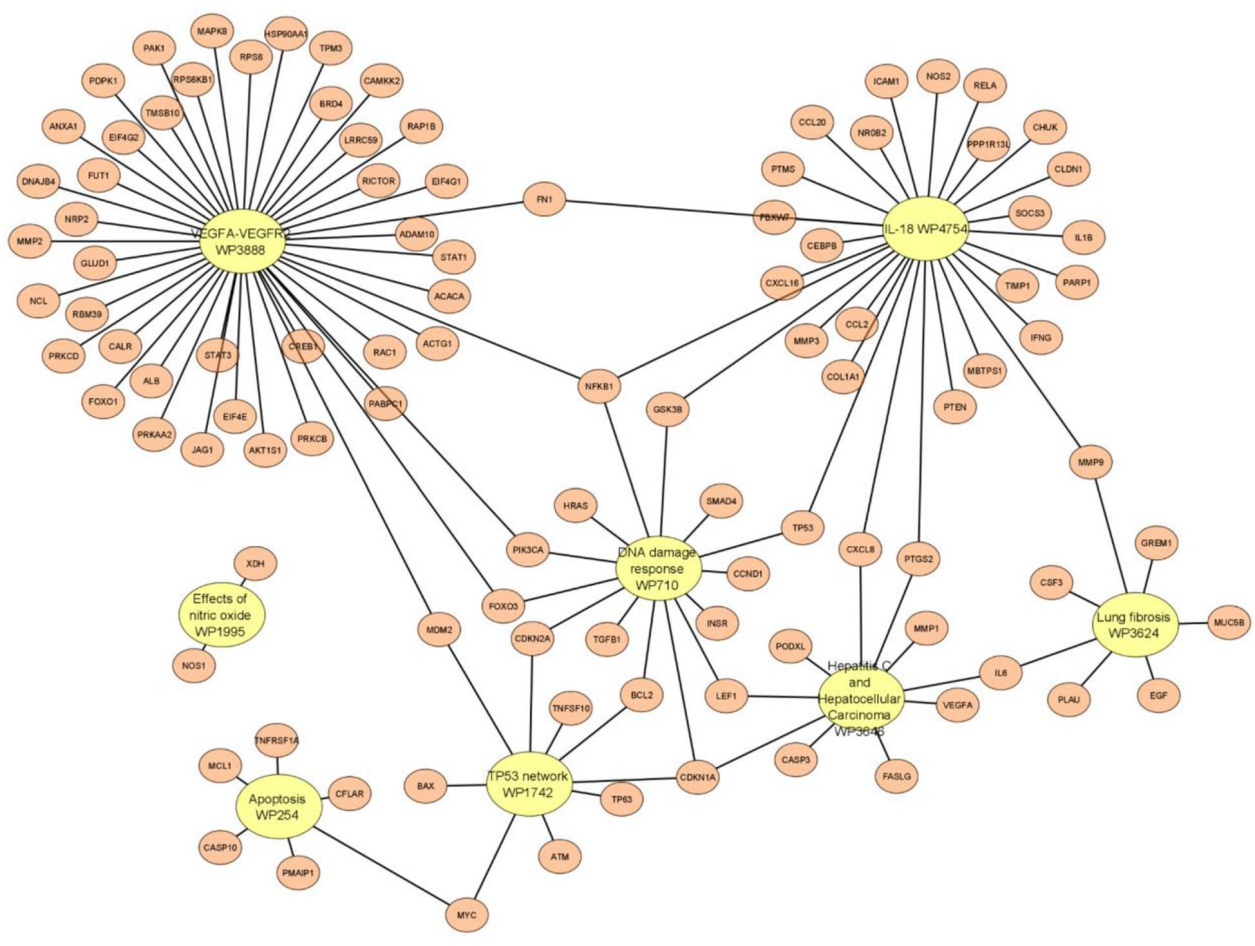
Network visualization of the gene interaction for the cell signaling pathway with overlapping genes for the ten GEO datasets using the cytoscape tool.

Next, we used the cytoscape tool^48^ to visualize the correlation network between the cell signaling pathways with the overlapping genes for all the top enriched pathways from the previous step. In total, we took the most 10 significant pathways that were enriched among the 20 disease-gene group pairs to visualize. Figure 9 represents the signaling pathway networks with overlapping genes across different GEO datasets.

**Figure 10:**
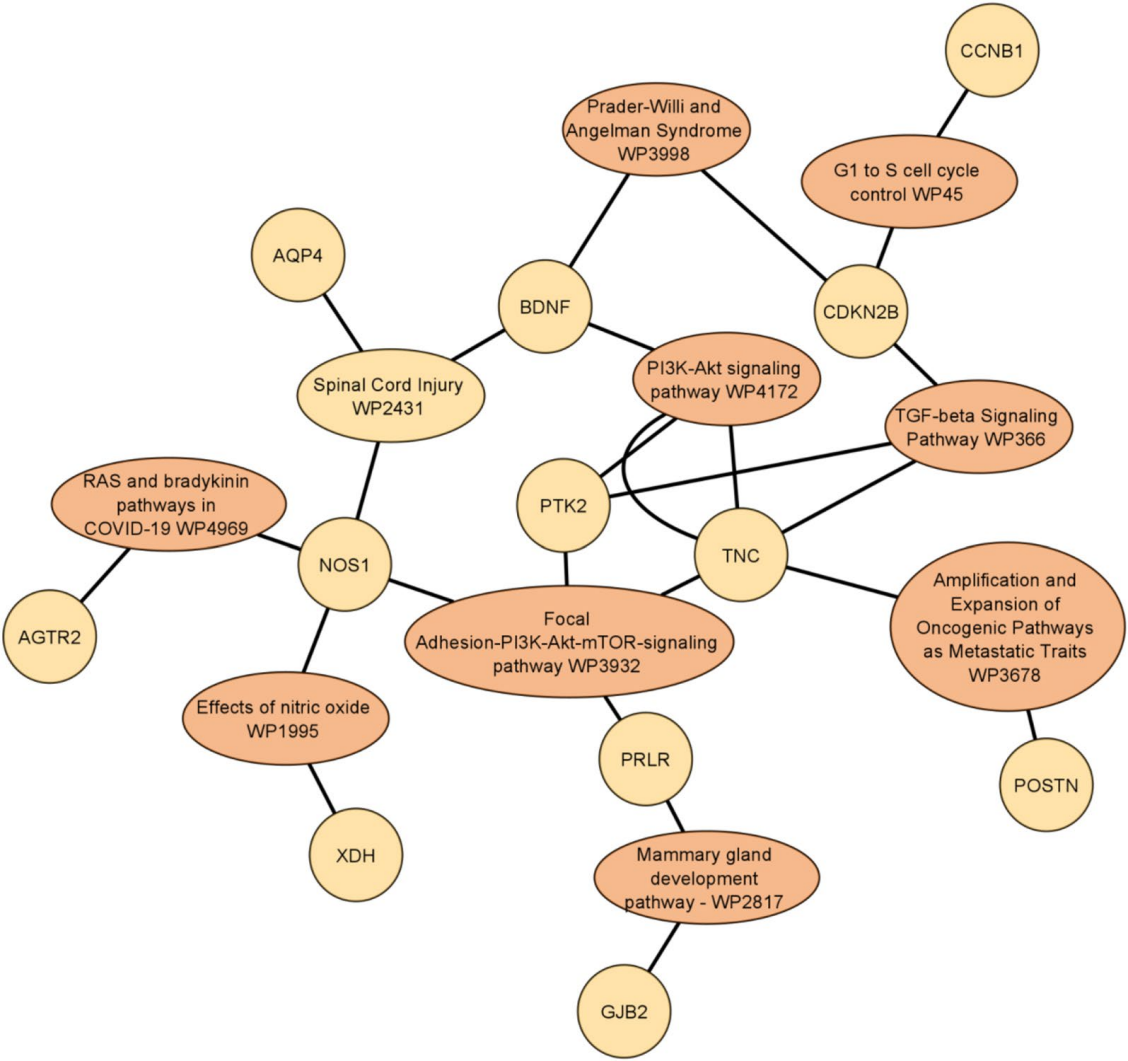
Network visualization of the cell signaling pathway with overlapping genes for the GDS3257 dataset using the cytoscape tool.

As we have stated, we examine 10 different GEO gene expression datasets, studying mostly different diseases. Figure 9 illustrates the most significant pathways related to all given datasets, indicating that disease genes are correlated and associated even when studying different diseases. The network in Fig. 9 shows that GediNET discovers important biological information related to various diseases. Moreover, we have studied the significance of GediNET on the data GDS3257 by considering the top 2 significant diseases having 12 distinct genes. Figure 10 illustrates the network of the most significant pathways and their related genes.

### Disease-disease associations

We assume that a disease is represented by a set of genes. The simple approach for finding a disease-disease association is by applying different association indices that consider the number of shared genes between the two diseases. For example, one might use the Jaccard Simpson, Geometric, Cosine, and even Pearson correlation coefficient (PCC)^32,33^.

Recently, different efforts toward Disease-Disease associations (DDA) are gaining attention for their importance in exploring novel associations of diseases and enhancing knowledge of disease relationships, which could further improve approaches to disease diagnosis, prognosis, and treatment. Yet, shared genes offer only limited information about the relationship between two diseases.

The number of known DDA and reliable associations is very small. Thus, it suggests that more efforts are required for DDA detections.

Disease-disease relationships through the incomplete human interactome^49^ are computational approaches that derive mathematical conditions for the identifiability of disease modules and show that the network-based location of each disease module determines its pathobiological relationship to other diseases. Suratanee A, Plaimas K.^50^ have developed a novel network-based scoring algorithm called DDA to identify the relationships between diseases in a large-scale study. Their method is developed based on a random walk prioritization in a protein–protein interaction network.

DisGeNET provides through its API, disease-disease associations that have been obtained by computing the number of shared genes and shared variants between pairs of diseases by source. DisGeNet uses two metrics to compute the DDA. The first one is the Jaccard Index (JI)$Jaccard_{G}=\frac{G_1 \cap G_2}{G_1 \cup G_2}$ , G_1_ is the set of genes associated with Disease 1, and G_2_ is the set of genes related to Disease 2.

The second one is Jaccard variance $Jaccard_{V}=\frac{V_1 \cap V_2}{V_1 \cup V_2}$, V_1_ is the set of variants associated with Disease 1, and V_2_ is the set of variants associated with Disease 2.

**Figure 11:**
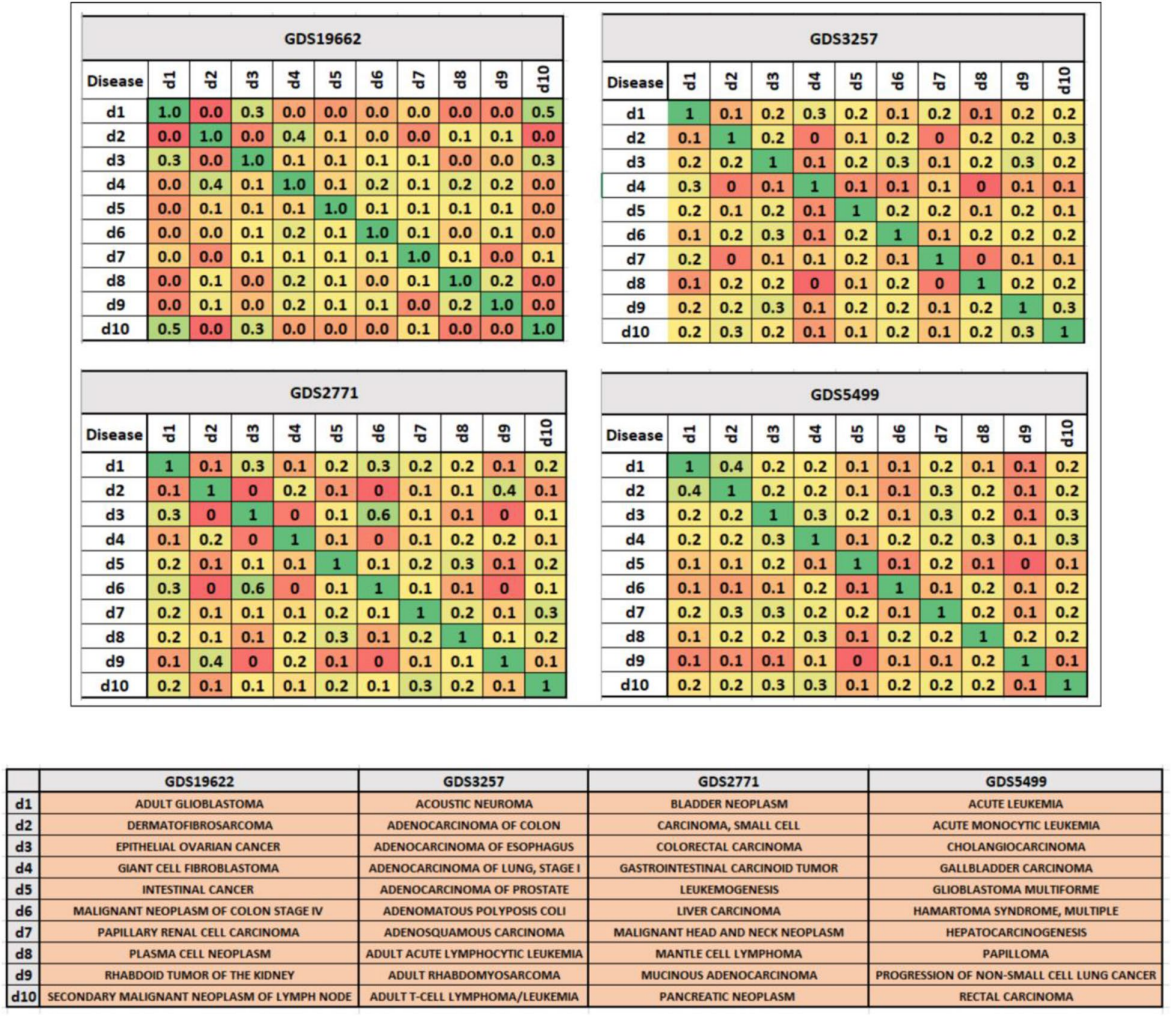
An example of the DDA for four datasets in GediNET. The number of shared genes for the top-scored disease group is represented. The upper panel shows the DDA for GDS1962, GDS3257, GDS2771 and GDS5499 datasets. The lower panel shows the annotations used in the DDA illustration formation.

In order to compute for each dataset, the standard DDA in GediNET, we have computed the fraction of the number of shared genes for each pair of the top-scored disease group for 4 datasets as illustrated in Fig. 11.

GediNET differs from the tools mentioned above in that it is based on machine learning for detecting the relationships between diseases, DDAs, which detect novel and previously unknown associations. We conducted a further analysis to explore if GediNET can identify novel relationships between diseases using DisGeNET API.

Table 9 illustrates for each data set its three top detected diseases by DisGeNET API and the top 3 ranked diseases by GediNET. For each detected disease by DisGeNet we have looked up the disease in the list of ranked diseases by GediNET to examine the two tools.

**Table 9:** Illustrates the three top detected diseases by DisGeNET API and the top 3 ranked diseases by GediNET for each GEO dataset.

GEO data set/target disease	The data disease	Top 1 disease name	Top 2 disease name	Top 3 disease name
GDS1962/brainstem glioblastoma	DisGeNET	Recurrent endometrial cancer (#193, pv = 0.16)	Adult astrocytic tumor (#253, pv = 0.22)	Alpha-thalassemia/mental retardation syndrome, nondeletion type, x-linked
	GediNET	Papillary renal cell carcinoma	Plasma cell neoplasm	Adult glioblastoma
GDS2545/metastatic prostate cancer	DisGeNET	Metastasis from malignant tumor of prostate (#25, pv = 0.01)	Hormone refractory prostate cancer (#274, pv = 0.34)	Secondary malignant neoplasm of bone (#62, pv = 0.04)
	GediNET	Childhood rhabdomyosarcoma	Rhabdomyosarcoma	Secondary malignant neoplasm of liver
GDS2771/lung cancer	DisGeNET	Primary malignant neoplasm of lung (#50, pv = 0.03)	Carcinoma of lung (#97, pv = 0.08)	Non-small cell lung carcinoma (#141, pv = 0.14)
	GediNET	Mantle cell lymphomA	Gastrointestinal carcinoid tumor	Mucinous adenocarcinoma
GDS3257/lung adenocarcinoma	DisGeNET	Non-small cell lung cancer recurrent (#116, pv = 0.11)	Adenosquamous cell lung cancer (#137, pv = 0.15)	Adenocarcinoma, metastatic (#200, 0.22)
	GediNET	Acoustic neuroma	Adenocarcinoma of colon	Adenocarcinoma of esophagus
GDS4206/Pediatric acute leukemia patients with early relapse: white blood cells	DisGeNET	Childhood leukemia (#96, pv = 0.13)	Melanoma (#29, pv = 0.03)	Glioblastoma multiforme (#115, pv = 0.18)
	GediNET	Acute leukemia	Adult diffuse large b-cell lymphoma	Esophageal carcinoma
GDS5499/pulmonary hypertension	DisGeNET	Idiopathic pulmonary hypertension	Vascular diseases	Endothelial dysfunction
	GediNET	Cholangiocarcinoma	Hepatocarcinogenesis	Papilloma
GDS3837/Non-small cell lung carcinoma in female nonsmokers	DisGeNET	Primary malignant neoplasm of lung	Carcinoma of lung (#10, pv = 0.009)	Neoplasm metastasis
	GediNET	Early-stage breast carcinoma	Meningioma, benign, no icd-o subtype	Colorectal carcinoma
GDS4516_4718/colorectal carcinoma	DisGeNET	Malignant neoplasm of colon and/or rectum (#3, pv = 0.002)	Carcinogenesis	Neoplasm metastasis
	GediNET	Acute leukemia	Acute lymphocytic leukemia	Malignant neoplasm of colon and/or rectum
GDS2547/metastatic prostate cancer	DisGeNET	Metastasis from malignant tumor of prostate (#27, pv = 0.02)	Hormone refractory prostate cancer (#91, pv = 0.1)	Secondary malignant neoplasm of bone (#123, pv = 0.18)
	GediNET	Malignant neoplasm of lung	Carcinoma of bladder	prostate carcinoma
GDS3268/ulcerative colitis	DisGeNET	Crohn disease	Inflammatory bowel diseases	Colitis
	GediNET	Malignant neoplasm of thyroid	Adenomatous polyposis coli	Leukemia, myelocytic, acute

For each detected disease by DisGeNET, we have looked up the disease in the list of robust ranked aggregated disease results by GediNET. The values in parenthesis for the rows of DisGeNET are the position of the disease and the P-value assigned by GediNET.

In Table 9 we have included additional information, the values in parenthesis for the rows of DisGeNET are the position of the disease and the P-value assigned by GediNET. Interestingly, excluding just one disease all the top three significant diseases detected by GediNET are novel. This suggests that the tool detects a new biological knowledge that the biology researcher should consider.

## Discussion and conclusion

In this study, we describe a novel approach for discovering disease-disease associations and detecting the genes/biomarkers associated with those diseases.

The approach is based on grouping the genes by their disease associations and then scoring those groups in terms of classification significance to train the machine learning model. For example, if a model created from the given data associated with a specific disease, such as lung cancer, is also found to apply to a subset of different diseases, this could suggest a previously undetected biological relationship with those other diseases that could inform clinical approaches not previously considered. The traditional approach of searching for genes that could be used as a biomarker in most cases yields a list of significant genes that solve the computational problem and does not take into account any prior knowledge about those genes, as such, their association with other diseases or even with other biological knowledge such as microRNA targets (see maTE tool^27^), or Pathways (See CogNet tool^28^), GeneOntology (See tool^32^).

### Potential limitations and future plans

The novelty of the GediNET approach lies in the fact that it scores gene groups by considering the contribution of all its members. One potential limitation of this approach that might be considered, is whether some members (genes) within a group may have a noisy impact and as a result adversely affect the overall classification performance. Other feature selection approaches that consider each gene individually, will not have this problem. However, to avoid this, we used a statistical t-test on the training dataset to first detect the top differentially expressed genes. The top 2000 differentially expressed genes were then used to extract the training datasets that were used as input to the G component. Thus, GediNET will always be dealing with the least noisy genes. One direction of future work is to perform internal gene scoring for each gene group to consider only those genes with the highest scores (Supplementary table S1).

Another potential limitation of our approach is the possibility that the size of the (gene) group could influence the performance. For example, by influencing Scoring component. Groups that contain larger numbers of gene would tend to have higher scores. This issue might be solved by considering a fixed number of representative genes from each group. An area of feature selection or feature ranking (scoring) that we have not addressed in this study, is the possibility that two groups of features that are useless when considered separately can be useful when they are combined. In GediNET, the scoring component treats each group individually. One potential future approach would be to develop the S component to score groups simultaneously to address this possibility.

Our GediNET tool is unique in that: (1) the search for the significant biomarkers/genes focuses on gene groups rather than single genes associated with the disease and (2) the final list of genes can be used to define new disease-disease associations as presented in Fig. 2, right panel. GediNET identifies important relationships between diseases, using DDA based machine learning, which explores novel associations that can enhance our knowledge of disease relationships and which could further improve approaches to disease diagnosis, prognosis, and treatment by detecting new relationship between diseases.

## Supplementary Information

Supplementary Tables.

## Data Availability

The datasets generated during and/or analyzed during the current study are available in the GEO (https://www.ncbi.nlm.nih.gov/geo/). The GediNET KNIME workflow can be downloaded from: https://github.com/malikyousef/GediNET.git or https://kni.me/w/3kH1SQV_mMUsMTS.
