# Supplementary material for: GediNET for discovering gene associations across diseases using knowledge based machine learning approach
Source: Sci Rep. 2022 Nov 19;12:19955. doi: 10.1038/s41598-022-24421-0 (PMC9675776; doi:10.1038/s41598-022-24421-0)
Supplement: Supplementary file 1 — Supplementary Tables. [file 41598_2022_24421_MOESM1_ESM.docx]

**Supplementary Data**

| **GDS1962** | | | | | | | | |
| --- | --- | --- | --- | --- | --- | --- | --- | --- |
| #Clusters | #Genes (Mean) | Accuracy (Mean) | Sensitivity (Mean) | Specificity (Mean) | F-measure (Mean) | Area Under Curve (Mean) | Recall (Mean) | Precision (Mean) |
| 10.00 | 136.74 | 0.93 | 0.93 | 0.92 | 0.95 | 0.98 | 0.93 | 0.97 |
| 9.00 | 127.68 | 0.93 | 0.93 | 0.92 | 0.95 | 0.98 | 0.93 | 0.97 |
| 8.00 | 116.02 | 0.93 | 0.94 | 0.92 | 0.95 | 0.98 | 0.94 | 0.97 |
| 7.00 | 111.16 | 0.93 | 0.93 | 0.91 | 0.95 | 0.98 | 0.93 | 0.97 |
| 6.00 | 102.02 | 0.93 | 0.94 | 0.92 | 0.95 | 0.98 | 0.94 | 0.97 |
| 5.00 | 92.88 | 0.93 | 0.93 | 0.93 | 0.95 | 0.98 | 0.93 | 0.97 |
| 4.00 | 78.37 | 0.93 | 0.93 | 0.92 | 0.95 | 0.98 | 0.93 | 0.97 |
| 3.00 | 62.47 | 0.93 | 0.94 | 0.92 | 0.95 | 0.98 | 0.94 | 0.97 |
| 2.00 | 45.57 | 0.93 | 0.93 | 0.93 | 0.95 | 0.97 | 0.93 | 0.98 |
| 1.00 | 21.61 | 0.92 | 0.93 | 0.92 | 0.94 | 0.96 | 0.93 | 0.97 |

**Supplementary Table S1:** GediNET performance for GDS1962 dataset for the top 2 groups. All values are the results of an average of 100-MCCV while considering the AUC for presenting the performance. The table shows the GEO accession in the first column, the number of genes in column #Genes while ACC is the accuracy, SEN is the sensitivity, SPE is the specificity, and the AUC is the area under the curve

| **GDS2545** | | | | | | | | |
| --- | --- | --- | --- | --- | --- | --- | --- | --- |
| #Clusters | #Genes (Mean) | Accuracy (Mean) | Sensitivity (Mean) | Specificity (Mean) | F-measure (Mean) | Area Under Curve (Mean) | Recall (Mean) | Precision (Mean) |
| 10.00 | 293.29 | 0.73 | 0.73 | 0.73 | 0.74 | 0.81 | 0.73 | 0.77 |
| 9.00 | 281.88 | 0.73 | 0.73 | 0.72 | 0.74 | 0.82 | 0.73 | 0.76 |
| 8.00 | 265.31 | 0.73 | 0.72 | 0.74 | 0.74 | 0.81 | 0.72 | 0.77 |
| 7.00 | 248.17 | 0.73 | 0.73 | 0.73 | 0.73 | 0.82 | 0.73 | 0.76 |
| 6.00 | 228.73 | 0.73 | 0.73 | 0.74 | 0.74 | 0.82 | 0.73 | 0.77 |
| 5.00 | 211.79 | 0.73 | 0.72 | 0.74 | 0.73 | 0.82 | 0.72 | 0.77 |
| 4.00 | 185.19 | 0.72 | 0.72 | 0.73 | 0.73 | 0.81 | 0.72 | 0.76 |
| 3.00 | 153.80 | 0.73 | 0.73 | 0.73 | 0.73 | 0.81 | 0.73 | 0.77 |
| 2.00 | 113.76 | 0.73 | 0.72 | 0.74 | 0.73 | 0.81 | 0.72 | 0.77 |
| 1.00 | 74.26 | 0.72 | 0.71 | 0.73 | 0.72 | 0.80 | 0.71 | 0.76 |

**Supplementary Table S2:** GediNET performance for GDS2545 dataset for the top 2 groups. All values are the results of an average of 100-MCCV while considering the AUC for presenting the performance. The table shows the GEO accession in the first column, the number of genes in column #Genes while ACC is the accuracy, SEN is the sensitivity, SPE is the specificity, and the AUC is the area under the curve

| **GDS2771** | | | | | | | | |
| --- | --- | --- | --- | --- | --- | --- | --- | --- |
| #Clusters | #Genes (Mean) | Accuracy (Mean) | Sensitivity (Mean) | Specificity (Mean) | F-measure (Mean) | Area Under Curve (Mean) | Recall (Mean) | Precision (Mean) |
| 10.00 | 231.84 | 0.64 | 0.70 | 0.58 | 0.67 | 0.70 | 0.70 | 0.66 |
| 9.00 | 220.06 | 0.64 | 0.70 | 0.58 | 0.67 | 0.70 | 0.70 | 0.66 |
| 8.00 | 209.01 | 0.65 | 0.70 | 0.59 | 0.67 | 0.70 | 0.70 | 0.67 |
| 7.00 | 194.77 | 0.64 | 0.70 | 0.59 | 0.67 | 0.70 | 0.70 | 0.66 |
| 6.00 | 183.21 | 0.64 | 0.69 | 0.59 | 0.67 | 0.70 | 0.69 | 0.66 |
| 5.00 | 164.00 | 0.65 | 0.70 | 0.59 | 0.67 | 0.69 | 0.70 | 0.66 |
| 4.00 | 146.79 | 0.64 | 0.69 | 0.59 | 0.66 | 0.69 | 0.69 | 0.66 |
| 3.00 | 128.91 | 0.65 | 0.69 | 0.60 | 0.67 | 0.70 | 0.69 | 0.67 |
| 2.00 | 97.83 | 0.64 | 0.69 | 0.59 | 0.67 | 0.70 | 0.69 | 0.66 |
| 1.00 | 59.79 | 0.64 | 0.67 | 0.60 | 0.66 | 0.68 | 0.67 | 0.66 |

**Supplementary Table S3:** GediNET performance for GDS2771 dataset for the top 2 groups. All values are the results of an average of 100-MCCV while considering the AUC for presenting the performance. The table shows the GEO accession in the first column, the number of genes in column #Genes while ACC is the accuracy, SEN is the sensitivity, SPE is the specificity, and the AUC is the area under the curve

| **GDS3257** | | | | | | | | |
| --- | --- | --- | --- | --- | --- | --- | --- | --- |
| #Clusters | #Genes (Mean) | Accuracy (Mean) | Sensitivity (Mean) | Specificity (Mean) | F-measure (Mean) | Area Under Curve (Mean) | Recall (Mean) | Precision (Mean) |
| 10.00 | 232.13 | 0.97 | 0.99 | 0.95 | 0.97 | 1.00 | 0.99 | 0.96 |
| 9.00 | 214.84 | 0.97 | 0.99 | 0.95 | 0.97 | 1.00 | 0.99 | 0.96 |
| 8.00 | 198.30 | 0.97 | 0.99 | 0.95 | 0.97 | 1.00 | 0.99 | 0.96 |
| 7.00 | 179.53 | 0.97 | 0.99 | 0.95 | 0.97 | 1.00 | 0.99 | 0.96 |
| 6.00 | 167.00 | 0.97 | 0.99 | 0.95 | 0.97 | 1.00 | 0.99 | 0.96 |
| 5.00 | 148.50 | 0.97 | 0.99 | 0.94 | 0.97 | 1.00 | 0.99 | 0.95 |
| 4.00 | 129.53 | 0.97 | 0.99 | 0.94 | 0.97 | 1.00 | 0.99 | 0.95 |
| 3.00 | 100.28 | 0.96 | 0.99 | 0.94 | 0.97 | 1.00 | 0.99 | 0.95 |
| 2.00 | 74.81 | 0.97 | 0.99 | 0.94 | 0.97 | 0.99 | 0.99 | 0.95 |
| 1.00 | 39.66 | 0.96 | 0.98 | 0.94 | 0.96 | 0.99 | 0.98 | 0.95 |

**Supplementary Table S4:** GediNET performance for GDS3257 dataset for the top 2 groups. All values are the results of an average of 100-MCCV while considering the AUC for presenting the performance. The table shows the GEO accession in the first column, the number of genes in column #Genes while ACC is the accuracy, SEN is the sensitivity, SPE is the specificity, and the AUC is the area under the curve

| **GDS3837** | | | | | | | | |
| --- | --- | --- | --- | --- | --- | --- | --- | --- |
| #Clusters | #Genes (Mean) | Accuracy (Mean) | Sensitivity (Mean) | Specificity (Mean) | F-measure (Mean) | Area Under Curve (Mean) | Recall (Mean) | Precision (Mean) |
| 10.00 | 265.00 | 0.92 | 0.83 | 1.00 | 0.91 | 0.96 | 0.83 | 1.00 |
| 9.00 | 223.00 | 0.92 | 0.83 | 1.00 | 0.91 | 0.94 | 0.83 | 1.00 |
| 8.00 | 214.00 | 0.92 | 0.83 | 1.00 | 0.91 | 0.94 | 0.83 | 1.00 |
| 7.00 | 209.00 | 0.92 | 0.83 | 1.00 | 0.91 | 0.97 | 0.83 | 1.00 |
| 6.00 | 209.00 | 0.92 | 0.83 | 1.00 | 0.91 | 0.97 | 0.83 | 1.00 |
| 5.00 | 194.00 | 0.92 | 0.83 | 1.00 | 0.91 | 1.00 | 0.83 | 1.00 |
| 4.00 | 191.00 | 0.92 | 0.83 | 1.00 | 0.91 | 0.94 | 0.83 | 1.00 |
| 3.00 | 191.00 | 0.92 | 0.83 | 1.00 | 0.91 | 0.94 | 0.83 | 1.00 |
| 2.00 | 21.00 | 0.92 | 0.83 | 1.00 | 0.91 | 0.92 | 0.83 | 1.00 |
| 1.00 | 6.00 | 0.92 | 0.83 | 1.00 | 0.91 | 0.86 | 0.83 | 1.00 |

**Supplementary Table S5:** GediNET performance for GDS3837 dataset for the top 2 groups. All values are the results of an average of 100-MCCV while considering the AUC for presenting the performance. The table shows the GEO accession in the first column, the number of genes in column #Genes while ACC is the accuracy, SEN is the sensitivity, SPE is the specificity, and the AUC is the area under the curve

| **GDS4206** | | | | | | | | |
| --- | --- | --- | --- | --- | --- | --- | --- | --- |
| #Clusters | #Genes (Mean) | Accuracy (Mean) | Sensitivity (Mean) | Specificity (Mean) | F-measure (Mean) | Area Under Curve (Mean) | Recall (Mean) | Precision (Mean) |
| 10.00 | 154.66 | 0.66 | 0.30 | 0.82 | #NUM! | 0.60 | 0.30 | 0.43 |
| 9.00 | 151.00 | 0.66 | 0.29 | 0.82 | #NUM! | 0.61 | 0.29 | 0.44 |
| 8.00 | 146.11 | 0.66 | 0.30 | 0.82 | #NUM! | 0.60 | 0.30 | 0.43 |
| 7.00 | 138.99 | 0.66 | 0.31 | 0.82 | #NUM! | 0.60 | 0.31 | 0.44 |
| 6.00 | 132.02 | 0.66 | 0.30 | 0.82 | #NUM! | 0.61 | 0.30 | 0.42 |
| 5.00 | 123.28 | 0.65 | 0.30 | 0.80 | #NUM! | 0.60 | 0.30 | 0.42 |
| 4.00 | 112.76 | 0.65 | 0.30 | 0.81 | #NUM! | 0.60 | 0.30 | 0.41 |
| 3.00 | 98.73 | 0.65 | 0.28 | 0.82 | #NUM! | 0.59 | 0.28 | 0.43 |
| 2.00 | 83.00 | 0.66 | 0.30 | 0.82 | #NUM! | 0.58 | 0.30 | 0.45 |
| 1.00 | 52.96 | 0.65 | 0.30 | 0.81 | #NUM! | 0.58 | 0.30 | 0.42 |

**Supplementary Table S6:** GediNET performance for GDS4206 dataset for the top 2 groups. All values are the results of an average of 100-MCCV while considering the AUC for presenting the performance. The table shows the GEO accession in the first column, the number of genes in column #Genes while ACC is the accuracy, SEN is the sensitivity, SPE is the specificity, and the AUC is the area under the curve

| **GDS4516_4718** | | | | | | | | |
| --- | --- | --- | --- | --- | --- | --- | --- | --- |
| #Clusters | #Genes (Mean) | Accuracy (Mean) | Sensitivity (Mean) | Specificity (Mean) | F-measure (Mean) | Area Under Curve (Mean) | Recall (Mean) | Precision (Mean) |
| 10.00 | 143.43 | 1.00 | 1.00 | 1.00 | 1.00 | 1.00 | 1.00 | 1.00 |
| 9.00 | 136.89 | 1.00 | 1.00 | 1.00 | 1.00 | 1.00 | 1.00 | 1.00 |
| 8.00 | 129.37 | 1.00 | 1.00 | 1.00 | 1.00 | 1.00 | 1.00 | 1.00 |
| 7.00 | 120.72 | 1.00 | 1.00 | 1.00 | 1.00 | 1.00 | 1.00 | 1.00 |
| 6.00 | 109.62 | 1.00 | 1.00 | 1.00 | 1.00 | 1.00 | 1.00 | 1.00 |
| 5.00 | 94.47 | 1.00 | 1.00 | 1.00 | 1.00 | 1.00 | 1.00 | 1.00 |
| 4.00 | 77.06 | 1.00 | 1.00 | 1.00 | 1.00 | 1.00 | 1.00 | 1.00 |
| 3.00 | 56.45 | 1.00 | 1.00 | 1.00 | 1.00 | 1.00 | 1.00 | 1.00 |
| 2.00 | 40.72 | 1.00 | 1.00 | 0.99 | 1.00 | 1.00 | 1.00 | 1.00 |
| 1.00 | 18.66 | 0.99 | 0.99 | 0.98 | 0.99 | 1.00 | 0.99 | 0.99 |

**Supplementary Table S7:** GediNET performance for GDS4516_4718 dataset for the top 2 groups. All values are the results of an average of 100-MCCV while considering the AUC for presenting the performance. The table shows the GEO accession in the first column, the number of genes in column #Genes while ACC is the accuracy, SEN is the sensitivity, SPE is the specificity, and the AUC is the area under the curve

| **GDS5499** | | | | | | | | |
| --- | --- | --- | --- | --- | --- | --- | --- | --- |
| #Clusters | #Genes (Mean) | Accuracy (Mean) | Sensitivity (Mean) | Specificity (Mean) | F-measure (Mean) | Area Under Curve (Mean) | Recall (Mean) | Precision (Mean) |
| 10.00 | 190.59 | 0.91 | 0.96 | 0.78 | 0.93 | 0.96 | 0.96 | 0.91 |
| 9.00 | 181.68 | 0.90 | 0.96 | 0.77 | 0.93 | 0.96 | 0.96 | 0.91 |
| 8.00 | 172.65 | 0.90 | 0.96 | 0.76 | 0.93 | 0.96 | 0.96 | 0.91 |
| 7.00 | 160.76 | 0.91 | 0.97 | 0.77 | 0.94 | 0.96 | 0.97 | 0.91 |
| 6.00 | 148.78 | 0.90 | 0.96 | 0.78 | 0.93 | 0.96 | 0.96 | 0.91 |
| 5.00 | 133.73 | 0.90 | 0.96 | 0.77 | 0.93 | 0.95 | 0.96 | 0.91 |
| 4.00 | 116.46 | 0.90 | 0.96 | 0.78 | 0.93 | 0.95 | 0.96 | 0.91 |
| 3.00 | 101.20 | 0.90 | 0.96 | 0.78 | 0.93 | 0.96 | 0.96 | 0.91 |
| 2.00 | 80.23 | 0.90 | 0.96 | 0.77 | 0.93 | 0.95 | 0.96 | 0.91 |
| 1.00 | 18.66 | 0.99 | 0.99 | 0.98 | 0.99 | 1.00 | 0.99 | 0.99 |

**Supplementary Table S8:** GediNET performance for GDS5499 dataset for the top 2 groups. All values are the results of an average of 100-MCCV while considering the AUC for presenting the performance. The table shows the GEO accession in the first column, the number of genes in column #Genes while ACC is the accuracy, SEN is the sensitivity, SPE is the specificity, and the AUC is the area under the curve.

| **GDS2547** | | | | | | | | |
| --- | --- | --- | --- | --- | --- | --- | --- | --- |
| #Clusters | #Genes (Mean) | Accuracy (Mean) | Sensitivity (Mean) | Specificity (Mean) | F-measure (Mean) | Area Under Curve (Mean) | Recall (Mean) | Precision (Mean) |
| 10.00 | 193.04 | 0.77 | 0.78 | 0.76 | 0.77 | 0.85 | 0.78 | 0.78 |
| 9.00 | 187.22 | 0.78 | 0.78 | 0.78 | 0.77 | 0.85 | 0.78 | 0.79 |
| 8.00 | 180.22 | 0.78 | 0.78 | 0.78 | 0.77 | 0.85 | 0.78 | 0.79 |
| 7.00 | 172.77 | 0.77 | 0.77 | 0.78 | 0.77 | 0.84 | 0.77 | 0.79 |
| 6.00 | 163.12 | 0.77 | 0.76 | 0.78 | 0.77 | 0.84 | 0.76 | 0.79 |
| 5.00 | 153.37 | 0.77 | 0.77 | 0.77 | 0.77 | 0.84 | 0.77 | 0.78 |
| 4.00 | 138.98 | 0.77 | 0.79 | 0.76 | 0.77 | 0.84 | 0.79 | 0.78 |
| 3.00 | 122.21 | 0.76 | 0.77 | 0.76 | 0.76 | 0.84 | 0.77 | 0.77 |
| 2.00 | 102.49 | 0.76 | 0.77 | 0.76 | 0.76 | 0.83 | 0.77 | 0.77 |
| 1.00 | 67.53 | 0.74 | 0.75 | 0.73 | 0.74 | 0.81 | 0.75 | 0.75 |

**Supplementary Table S9:** GediNET performance for GDS2547 dataset for the top 2 groups. All values are the results of an average of 100-MCCV while considering the AUC for presenting the performance. The table shows the GEO accession in the first column, the number of genes in column #Genes while ACC is the accuracy, SEN is the sensitivity, SPE is the specificity, and the AUC is the area under the curve.

| **GDS3268** | | | | | | | | |
| --- | --- | --- | --- | --- | --- | --- | --- | --- |
| #Clusters | #Genes (Mean) | Accuracy (Mean) | Sensitivity (Mean) | Specificity (Mean) | F-measure (Mean) | Area Under Curve (Mean) | Recall (Mean) | Precision (Mean) |
| 10.00 | 211.24 | 0.67 | 0.72 | 0.63 | 0.70 | 0.75 | 0.72 | 0.69 |
| 9.00 | 204.29 | 0.68 | 0.73 | 0.62 | 0.70 | 0.75 | 0.73 | 0.69 |
| 8.00 | 195.75 | 0.68 | 0.72 | 0.63 | 0.70 | 0.75 | 0.72 | 0.70 |
| 7.00 | 188.90 | 0.67 | 0.70 | 0.64 | 0.69 | 0.74 | 0.70 | 0.70 |
| 6.00 | 180.75 | 0.67 | 0.70 | 0.64 | 0.69 | 0.75 | 0.70 | 0.69 |
| 5.00 | 168.01 | 0.67 | 0.71 | 0.63 | 0.69 | 0.74 | 0.71 | 0.70 |
| 4.00 | 156.06 | 0.68 | 0.70 | 0.66 | 0.70 | 0.74 | 0.70 | 0.71 |
| 3.00 | 140.03 | 0.68 | 0.70 | 0.66 | 0.70 | 0.75 | 0.70 | 0.71 |
| 2.00 | 115.70 | 0.67 | 0.70 | 0.63 | 0.69 | 0.74 | 0.70 | 0.69 |
| 1.00 | 70.88 | 0.67 | 0.68 | 0.65 | 0.68 | 0.73 | 0.68 | 0.70 |

**Supplementary Table S10:** GediNET performance for GDS3268 dataset for the top 2 groups. All values are the results of an average of 100-MCCV while considering the AUC for presenting the performance. The table shows the GEO accession in the first column, the number of genes in column #Genes while ACC is the accuracy, SEN is the sensitivity, SPE is the specificity, and the AUC is the area under the curve.
